# The Archbishop-Designate and the League of Mercy

**Published:** 1903-02-07

**Authors:** 


### THE ARCHBISHOP-DESIGNATE AND THE LEAGUE OF MERCY.

The Archbishop-Designate of Canterbury, Dr. Randall Davidson, having read Sir Henry Burdett's address just published, entitled "In the Days of Health," explaining the work of the League of Mercy, has spontaneously written : "May God's best blessing attend your devoted labour for the sick and suffering." Dr. Davidson's action will necessarily encourage and stimulate the efforts of the officers and members of the League in aid of the hospitals. Public interest in its work is everywhere increasing. At Paddington, on Thursday, although 1,800 people were crowded into the hall, the numbers seeking admission were still so great that the outer doors had to be forcibly closed. The new Archbishop's spontaneous action must tend to still further extend the movement and deepen the interest amongst all classes of Londoners in favour of the League.

